# A Single 30 Minutes Bout of Combination Physical Exercises Improved Inhibition and Vigor-Mood in Middle-Aged and Older Females: Evidence From a Randomized Controlled Trial

**DOI:** 10.3389/fnagi.2020.00179

**Published:** 2020-06-24

**Authors:** Rui Nouchi, Haruka Nouchi, Ryuta Kawashima

**Affiliations:** ^1^Department of Cognitive Health Science, Institute of Development, Aging and Cancer, Tohoku University, Sendai, Japan; ^2^Smart Aging Research Center, Tohoku University, Sendai, Japan; ^3^Department of Functional Brain Imaging, Institute of Development, Aging and Cancer, Tohoku University, Sendai, Japan

**Keywords:** acute effect, combination exercise, inhibition, vigor mood, older adults

## Abstract

**Background:**

Long-term combination of physical exercises has reported benefits for cognitive functions and mood states. However, it remains unclear whether a single bout of combination exercise training has acute positive effects on cognitive functions and mood states in middle-aged and older women. It is important to investigate acute effect of physical exercise because it would help to understand a mechanism of benefits of physical exercise. The purpose of this study was to investigate 30 min of a single bout of combination exercise training on cognition and mood states in middle-aged and older females.

**Methods:**

In this single-blinded randomized control trial (RCT), middle-aged and older females were assigned randomly to two groups: a combination exercise group and a no-exercise control group. The former group did the combination exercise training (aerobic, strength, and stretching exercises) for 30 min. Meanwhile, the latter group did not do any exercise and waited for 30 min. We measured cognitive functions and mood performance states before and after the exercise or control interventions.

**Results:**

Our main results demonstrated that, compared to the control group, the combination exercise improved inhibition (reverse Stroop and Stroop) and increased vigor–activity mood scores in both middle-aged and older groups. We also found that the only combination exercise group showed the significant positive correlations between improved inhibition performance and improved vigor–activity mood.

**Discussion:**

This randomized controlled trial revealed the acute benefits of combination exercise on inhibition in executive functions and vigor–activity in the healthy middle-aged and older females. Our results provided the scientific evidence related to acute effects of the single bout of the combination exercise training. It suggests that we would be better to do the 30 min physical exercise for our health.

**Clinical Trial Registration:**

This trial was registered in the University Hospital Medical Information Network Clinical Trials Registry (UMIN000029681). Registered 24 October 2017, https://upload.umin.ac.jp/cgi-bin/ctr/ctr_view_reg.cgi?recptno=R000033922.

## Introduction

Physical exercise has positive effects on cognitive function and moods. For example, observation studies using a meta-analysis reported a positive correlation between regular physical exercise and cognitive functions (Sofi et al., 2011). People who exercised regularly showed lower negative and higher positive mood. Consistent with these observational studies, long-term exercise intervention using randomized controlled trial (RCT) showed that aerobic and strength training led to improved cognitive functions (Bherer et al., 2013) and mood (Conn, 2010).

In addition to long-term exercise benefits, growing research has investigated the effects of acute exercise on cognitive functions and moods (Basso and Suzuki, 2016). The acute exercise studies compared the performance of cognitive functions (and mood states) immediately before and after a single bout of exercise. Meta-analysis and systematic review studies demonstrated that acute exercise improved cognitive performance (Chang et al., 2012; Ludyga et al., 2016; Levin et al., 2017) and changed mood states (Yeung, 1996; Elkington et al., 2017).

Although earlier studies showed that acute exercise is beneficial for cognitive functions and mood, there are several unresolved issues (e.g., age, types of exercise, outcome measures). A meta-analysis study for acute benefits of exercise reported that combined aerobic and strength exercises had a larger effect size on cognitive function compared to aerobic or strength exercise alone (Chang et al., 2012). However, previous studies mainly investigated the acute benefit of aerobic exercise training on cognition (Lambourne and Tomporowski, 2010; Chang et al., 2012; Verburgh et al., 2014). However, one systematic review for mood claimed that only one study investigated the acute benefit of the combination exercise on mood states (Elkington et al., 2017). Therefore, it is still unknown whether an acute combination exercise has benefits on cognitive functions and mood states.

Previous studies mentioned that exercise intensity would affect acute benefits of exercise on cognition and mood. For cognition, previous meta-analysis study demonstrated that compared to control condition, any intensity exercise have positive impacts on cognition (Chang et al., 2012). But, the previous meta-analysis did not separate aerobic and strength exercise. Recent meta-analysis studies suggested that compared to the control condition, high intensity exercise would have more beneficial effect on cognition in both aerobic (Moreau and Chou, 2019) and strength exercise (Wilke et al., 2019). Other studies showed that there were no significant differences of acute benefit between aerobic and strength exercise (Dunsky et al., 2017; Wilke et al., 2019). One study (Chang et al., 2017) directly compared the acute benefit of the high intensity aerobic exercise, the high intensity strength exercise, and the moderate intensity combination exercise on cognition. Interestingly, the result showed the moderate intensity combination exercise showed greater acute benefit on cognition compared to the high intensity aerobic and strength exercise (Chang et al., 2017). For mood, the exercise intensity differently influenced positive and negative mood. The high intensity aerobic exercise reduced negative mood such as anxiety. On the other hand, the light intensity aerobic exercise (Reed and Ones, 2006) and the moderate intensity resistant exercise (Bibeau et al., 2010) improved positive mood such as arousal. One study reported that the moderate intensity combination exercise training showed the acute benefits on positive mood (Maraki et al., 2005). Based on the previous studies, we hypothesized the moderate intensity combination exercise would have beneficial effect on both cognition and mood. Almost all acute exercise studies recruited young adults. One meta-analysis study for cognitive functions revealed that the average age of the participants in 61 studies was 28.51 years (SD = 17.21) (Chang et al., 2012). Among these, 42 studies used young adult population (20–30 years), 4 focused on middle-aged adults (30–60 years), and 6 on older adults (>60 years) (Chang et al., 2012). For assessing the benefits of acute exercise on mood states, few studies have recruited middle-aged and older adults (Yeung, 1996; Elkington et al., 2017). Therefore, it is not clear whether acute exercise has positive effects on cognition and mood in middle-aged and older adults.

Previous studies measured just one or two cognitive functional measures in executive functions) (Hyodo et al., 2012; Hogan et al., 2013; Byun et al., 2014; Ji et al., 2017; Legrand et al., 2018). Mainly, the executive functions are divided into three domains: inhibition, shifting, and updating (Miyake et al., 2000; Webb et al., 2018). However, previous studies used only one of the three executive functional domains, especially inhibition using the Stroop test (Hyodo et al., 2012; Hogan et al., 2013; Byun et al., 2014; Ji et al., 2017; Legrand et al., 2018). A meta-analysis study for aerobic exercise reported that there was no significant difference in the acute benefits of aerobic exercise on inhibition, shifting, and updating (Ludyga et al., 2016). However, it is still unclear whether acute combination exercises can improve specific cognitive functions or diverse cognitive functions.

To assess the abovementioned issues, we conducted a randomized controlled trial. The purpose of this study was to investigate the benefits of acute exercise using a combination exercise on diverse cognitive functions and mood states in healthy middle-aged and older female adults. In this study, the primary measures were cognitive functions. The secondary measures were mood states. We chose the moderate intensity combination exercise constituting aerobic, strength, and stretching training because the previous long-term intervention study using the same combination exercise training showed positive benefits on a wide range of cognitive functions (Nouchi et al., 2014). We recruited middle-aged and older adults and measured a variety of cognitive functions: processing speed, executive functions (inhibition, shifting, and updating), and attention.

Moreover, we recruited only the female participants from an exercise gym because sex and initial fitness level affected benefits from acute exercises. Sex differences in physical ability existed (Courtright et al., 2013). Individuals retaining a high fitness level, and female participants showed more significant improvements in cognitive functions (Chang et al., 2012) and mood states (McDowell et al., 2016) after training. The sex difference was not our main research question. Therefore, we recruited older female adults. Previous studies suggested that possible underlying mechanisms of the sex difference are brain-derived neurotrophic factor (BDNF) and sex steroid hormones (estrogens) (Barha et al., 2017). Acute and long-term exercise increase the estrogens level in females (Janssen, 2016). The increased estrogens acts upregulate BDNF levels (Scharfman et al., 2003). The estrogen and BDNF has important roles of neural plasticity and cognitive improvements (Phillips et al., 2014; Triviño-Paredes et al., 2016). Therefore, females show greater benefits from physical exercise.

Based on the previous findings, we made the following hypotheses. For cognitive functions, the previous study demonstrated that the combination of exercise training improved inhibition performance in executive functions (Quintero et al., 2018). One meta-analysis of the acute exercise study reported that middle-aged and older adults showed a similar effect size on cognitive performance (Chang et al., 2012). Therefore, we assumed that the combination exercise training regime would improve inhibition performance in both middle-aged and older females. For mood states, the previous study using a combination of exercise training increased the positive mood (Maraki et al., 2005). Besides, acute aerobic exercise improved high arousal and positive mood, including activation, elevation, and enthusiasm, equally in middle-aged and older adults (Hogan et al., 2013). Therefore, we hypothesized that the combination exercise training regime would improve high arousal mood, such as Vigor in Profile of Mood States (POMS).

## Method

### Randomized Controlled Trial Design and Setting of This Trial

This RCT was conducted in Sendai city, Japan, and the Ethics Committee of the Tohoku University Hospital approved the study protocol. The RCT was registered in the University Hospital Medical Information Network (UMIN) Clinical Trial Registry (UMIN000029681).

To assess the effect of the combination exercise on cognitive functions in healthy middle-aged and older women, we conducted a single-blinded RCT. Testers were blinded to the study hypothesis and the group membership of participants. The primary outcome measure was the Stroop task performance in cognitive function. Other measurements, such as other cognitive functions and mood states, were the secondary outcome measures. The Consolidated Standards of Reporting Trials (CONSORT) statement^1^ (see Supplementary Table S1) was used to report the study structure.

**FIGURE 1:**
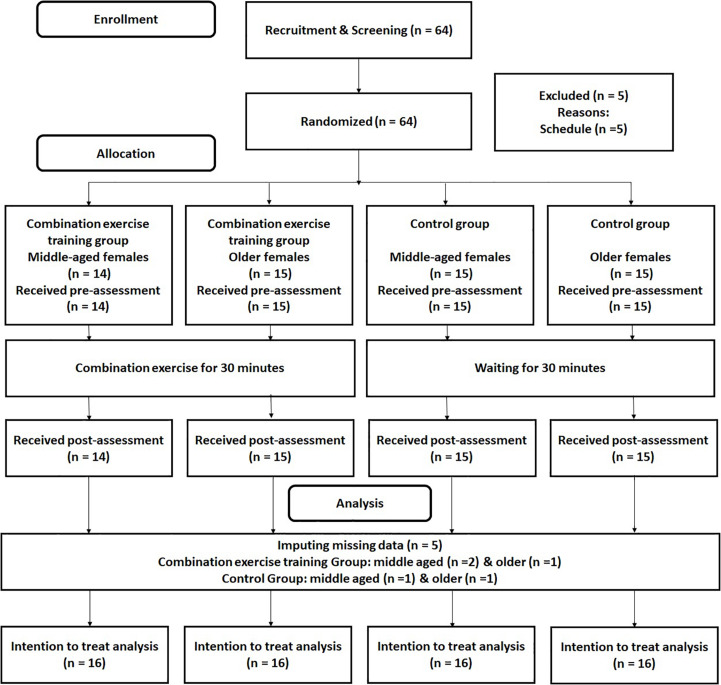
CONSORT diagram.

The RCT design is presented in Figure 1. Participants took pre-assessments for cognitive functions and mood states. After 5 min rest, participants did a combination exercise or a no-exercise control intervention for 30 min. After 5 min rest, they received took pos- assessments for cognitive function and mood states. In the pre and post assessments, participants firstly answered their mood states by POMS, then they received cognitive functions tests. To reduce of the order effect, the order of cognitive function tests was randomized among participants. In addition, to keep the time gap between pre and post tests, we used the same order of cognitive function tests in the pre and post assessments in each participant.

### Participants

Advertisements were used to recruit healthy middle-aged and older adults who were familiar with the training of combination exercises in Sendai city in Japan. We ran the advertisements at the entrance of the exercise gym for 2 weeks. Inclusion and exclusion criteria were printed on the flyers. The 64 interested participants contacted the research group by phone (Figure 1). All interested participants participated in an orientation meeting. At the meeting, one researcher (RN) explained the study details. We received informed consent from each participant. After that, the researcher also checked whether the interested participant was eligible to participate in this study. At that time, the 64 participants met the inclusion criteria. The 64 interested participants were assigned randomly to combination exercise or control groups. After the randomization, five participants were excluded because of their schedule. Based on the intention to treatment analysis (ITT), we did not recruit additional participants, but we imputed the missing data using multiple imputation methods (please see the section “Analysis”). Table 1 presents the baseline characteristics of all remaining participants [*n* = 59, average age = 62.29 (*SD* = 8.76)]. To check the group differences, we conducted 2 (group: combination exercise and control) by 2 (age category: middle age and older) analysis of variance (ANOVA). We found the significant main effect of age category on participants average age. It confirmed that there was the significant difference of age between the middle-aged and older age female group. However, we did not find any significant results for the main effects of group and the interaction between group and age category.

**TABLE 1:** Characteristics of participants.

	**Combination exercise group**				**Control group**				**2 (group) by 2 (age category) ANOVA**		
	**Middle age**		**Older**		**Middle age**		**Older**		***p*-Value**		
	**Mean**	**SD**	**Mean**	**SD**	**Mean**	**SD**	**Mean**	**SD**	**Group effect**	**Age-category effect**	**Interaction effect**
Age	55.00	3.59	69.73	5.32	54.88	3.74	70.07	5.38	0.96	0.00	0.75
Education	14.60	2.26	13.47	1.41	14.33	2.44	13.67	1.99	0.98	0.10	0.64
Height	158.03	4.12	155.25	3.78	158.99	3.60	154.66	3.62	0.89	0.00	0.38
Weight	55.66	4.48	55.09	4.34	54.45	3.91	54.99	4.63	0.30	0.44	0.66
BMI	22.35	2.86	23.90	2.21	21.61	2.05	23.16	2.16	0.31	0.02	0.96
%HRmax	71.54	3.05	71.36	2.35	–	–	–	–	–	–	–
**Cognitive functions at the baseline**											
Cd	90.21	8.16	71.67	15.71	90.06	12.89	69.93	16.88	0.99	0.00	0.76
rST	36.86	6.87	25.80	7.95	37.94	4.99	27.29	8.12	0.34	0.00	0.91
ST	36.71	7.00	24.60	9.35	37.88	5.03	27.00	8.30	0.34	0.00	0.67
LFT	10.43	2.87	9.00	1.89	8.75	2.84	9.14	3.74	0.36	0.58	0.30
Updating	11.36	2.37	8.73	2.76	10.44	1.90	9.36	3.63	0.89	0.02	0.42
D-CAT	201.50	37.47	171.13	33.23	196.81	24.91	162.07	27.38	0.70	0.00	0.57
**Mood at the baseline**											
AH	3.93	2.56	3.33	2.61	4.13	3.22	3.79	4.30	0.70	0.23	0.85
CB	3.86	2.11	4.20	2.34	5.25	3.34	4.71	4.03	0.24	0.99	0.63
DD	2.86	2.11	3.60	2.64	3.44	3.54	3.93	3.56	0.72	0.61	0.97
FI	4.71	3.79	4.00	1.85	4.81	3.21	4.64	3.59	0.88	0.78	0.56
TA	5.50	2.14	6.33	2.35	7.50	4.21	5.86	3.39	0.24	0.68	0.11
VA	7.79	2.97	9.47	2.85	8.69	3.40	8.79	4.28	0.58	0.16	0.22
F	11.71	3.41	12.53	2.36	12.13	3.03	11.64	4.75	0.92	0.34	0.30
TMD	24.79	10.53	24.53	10.22	28.56	17.56	25.79	17.40	0.54	0.97	0.81

*Unit of education is years, %HRmax, percentage of maximum heart rate; SD, standard deviation; Cd, digit symbol coding; rST, reverse Stroop task; ST, Stroop task; LFT, letter fluency task; Updating, working memory updating task; D-CAT, digit cancellation task; AH, Anger–Hostility; CD, Confusion–Bewilderment; DD, Depression–Dejection; FI, Fatigue–Inertia; TA, Tension–Anxiety; VA, Vigor–Activity; F, Friendliness; TMD, total mood disturbance. Means and SDs were the pooled means and SD after multiple imputations (*m* = 20).*

### Inclusion and Exclusion Criteria

Based on our earlier intervention studies (Nouchi et al., 2012b, 2014), we used the following inclusion and exclusion criteria: inclusion - (1) right-handed; (2) native Japanese speakers; (3) 40–80 years old; (4) a regular exercise habit meaning that they exercise at the gym at least once a week; (5) unconcerned about their own memory functions, not using medications known to interfere with cognitive functions (including benzodiazepines, antidepressants or other central nervous agents); (6) no history of any disease known to affect the central nervous system, including thyroid disease, multiple sclerosis, Parkinson disease, stroke, diabetes, and severe hypertension (systolic blood pressure is over 180, diastolic blood pressure is over 110). Also, participants joining other cognition-related intervention studies were excluded.

In the future, we will plan to investigate the neural correlates of a single physical exercise training on cognitive functions and moods based on this study. The typical neuroimaging studies recruited the only right-handed people due to effect of handedness on brain activities (Hatta, 2007). Therefore, we recruited the only ring-handed people in this study.

### Sample Size

We calculated the sample size using G^∗^Power software (Faul et al., 2009). The sample size was based on the change in the Stroop score because the primary outcome measure in this RCT was the cognitive functions. A study using the single bout of a moderate exercise reported large effect size (η^2^ = 0.28) on the Stroop task between exercise and no-intervention conditions (Hyodo et al., 2012).

Based on that evidence, we expected an effect size between medium and large (*f* = 0.36). To calculate the sample size, we set 2 (group: combination exercise and control groups) by 2 [age category: middle-aged (40–60 years) and older adults (60–80 years)] between an analysis of covariance (ANCOVA) model using a pre-intervention score of Stroop test and age as covariates, α = 0.05, and 0.80 power. Our first assumption is the main effect of group, which is the combination exercise group would show a greater benefit on the Stroop performance compared to the control group. The estimated sample size was 64; each condition had 16 participants.

### Randomization

To assign the 64 interested participants randomly to combination exercise and control groups, we used an online program for randomization^2^. RN conducted this randomization method. We stratified participants based on age category (middle-age and older adults). We used blocked randomization (block size: 4) with an allocation ratio of 1:1.

### Interventions

In the combination exercise group, developed by Curves, participants were asked to do the combination, which combined training of three types: aerobic, strength, and stretching (Nouchi et al., 2012b, 2014). Participants performed the combination exercise training for 30 min. The following descriptions in combination exercise training are mostly reproduced from our earlier report (Nouchi et al., 2014).“Each circuit-style workout consists of 12 strength training exercises (chest press/seated row, squat, shoulder press/lat pull, leg extension/leg curl, abdominal crunch/back extension, lateral lift, elbow flexion/extension, horizontal leg press, pectoral deck, oblique, hip abductor/adductor, gluteus). The strength training machines included calibrated pneumatic resistance pistons that allowed for opposing muscle groups to be trained in a concentric-only fashion. Participants were informed of the proper use of all equipment and were instructed to complete as many repetitions in a 30-s period. In a continuous interval fashion, participants performed floor-based aerobic training (e.g., running/skipping in place, and arm circles) on recovery pads for a 30-s period after each resistance exercise to maintain a consistent exercise heart rate corresponding to 60–80% of their maximum heart rate. All workouts were supervised by trained exercise instructors who assisted with proper exercise technique and maintenance of adequate exercise intensity. Participants must complete two circuits (24 min). After two rotations, participants did standardized whole-body stretching training (6 min). The whole-body stretching training consists of 12 stretching exercises (Achilles’ tendon, sole, thigh, armpit, shoulder, shoulder/upper arm, chest/arm, shoulder/chest/arm, waist, back of the knee, the base of the thigh, back).” In order to monitor the exercise intensity, the participant’s heart rate was measured by Polar H10 during the combination exercise training regime (Himmelmeier et al., 2019). Then, using age-predicted maximum heart rate using (220-age), we calculated the percentage of maximal heart rate (%HRmax) during the combination exercise.

For the control group, participants were asked to sit on a chair and wait for 30 min.

### Cognitive Functional Measures

To investigate the effect of combination exercise on cognitive functions, we assessed the performance of processing speed, executive functions (inhibition, shifting, and updating), and attention. About 30 min were necessary to conduct all cognitive tests.

To assess processing speed, we used digit symbol coding (Cd) from WAIS-III (Wechsler, 1997). The following descriptions in Cd are mostly reproduced from our earlier report (Nouchi et al., 2012a). “For Cd, the participants were shown a series of symbols that were paired with numbers. Using a key within a 120 s time limit, participants draw each symbol under its corresponding number. The primary measure of this test was the number of correct answers.”

We measured inhibition, shifting, and updating performance in executive functions. We used a Stroop task (ST) and a reverse Stroop task (rST) for the inhibition performance (Hakoda and Watanabe, 2004) and verbal fluency task for shifting performance (Ito et al., 2004). “In the ST, in the leftmost of six columns, a word naming a color was printed in another color (e.g., ‘red’ is printed in blue letters); the other five columns contain words naming colors. Participants must check the column containing the word naming the color of the word in the leftmost column. In the reverse ST, in the leftmost of six columns, a word naming a color was printed in another color (e.g., ‘red’ is printed in blue letters); the other five columns were filled respectively with five different colors from which participants must check the column with the color matching the written word in the leftmost column. In each task, participants were instructed to complete as many of these exercises as possible in 1 min. The primary measure for this task was the number of correct items” (Nouchi et al., 2012a).

For the shifting performance, we used a verbal fluency task (Ito et al., 2004). This study used the Japanese version of the letter fluency task (LFT). Participants were asked to generate as many words as possible beginning with a specific letter [Japanese word “KA”) in 1 min (Ito et al., 2004)]. The primary measure was the total number of generated words.

To measure updating performance, we used a working memory updating task (Kato, 2006). In this task, participants were asked to remember the last three digits (3-digit condition) and the last four digits of a list (4-digit condition). The order of the condition was fixed, with the 3-digit condition coming first. For example, in a 3-digit condition, participants listened to the digit list (e.g., 9-6-3-4-2). Then, they wrote down the last three digits of the list (e.g., 3-4-2). The list length for the 3-digit condition was from 3 to 9. The list length for the 4-digit condition was from 4 to 10. Each condition had eight lists (total 16 lists). The primary outcome measure was the total number of correct answers in each digit-condition (Max = 16).

To check the attention performance, we used the digit cancellation task (D-CAT) (Hatta et al., 2000). “The test sheet consists of 12 rows of 50 digits. Each row contains five sets of numbers 0–9 arranged in random order. Consequently, any single digit would appear five times in each row with randomly determined neighbors. D-CAT consists of three such sheets. Participants were instructed to search for the target number(s) that had been specified to them and to delete each one with a slash mark as quickly and as accurately as possible until the experimenter sent a stop signal” (Nouchi et al., 2013). We asked the participants to delete three digits (8, 3, and 7) in 1 min. It was emphasized that all instructed target numbers should be canceled without omission. The primary measure of this test is the number of hits (correct answers).

### Mood State Measure

To ascertain the change of mood state, we used a short version Profile of Mood State Second Edition (POMS2) (Heuchert and McNair, 2012; Yokoyama and Watanabe, 2015). We measured mood states using Profile of Mood States (POMS) (Heuchert and McNair, 2012; Yokoyama and Watanabe, 2015) because (1) the POMS can measure acute mood changes (Yokoyama and Watanabe, 2015), (2) it was suitable to measure mood in middle and older adults (Gibson, 1997), and (3) most previous acute exercise studies had employed it (Yeung, 1996). The following descriptions in POMS2 are mostly reproduced from our earlier report (Nouchi et al., 2019). POMS2 has seven subscales with five-point scales (total of 35 items).” POMS2 can measure mood states for the prior week of Tension–Anxiety (TA), Depression–Dejection (DD), Anger–Hostility (AH), Vigor–Activity (VA), Fatigue–Inertia (FI), Confusion–Bewilderment (CB), and Friendliness (F).” We also calculated total mood disturbance (TMD) score [total score of negative mood subscale (TA + DD + AH + FI + CB) minus VA score], which represents general mood states.

### Analysis

All participants were included based on the intention to treat (ITT) principle. We used the changed scores in each score (post-score minus pre-score). Then, we imputed missing values using the predictive mean matching because the predictive mean matching method in the multiple imputation can work even if a sample size was small (Kleinke, 2018). All variables of the pre-, post-, and change scores and participants’ age were included in the data imputation process (*m* = 20). We used “mice” in the mice package (van Buuren and Groothuis-Oudshoorn, 2011). Finally, we analyzed the 20 imputed dataset using 2 (group: combination exercise and control) by 2 (age category: middle age and older) analysis of covariance (ANCOVA) with permutation tests. We used permutation tests because they are suitable for small sample analysis and are distributed freely. In ANCOVA, the changed score is the dependent variable. The group and age are the independent variables. The pre-scores in the dependent variable and age were used as covariates. The permutation ANCOVAs were conducted using “aovp” in the lmPerm package^3^. Finally, we pooled the *F* values from 20 data sets. The analysis procedure was the same as the previous study (Nouchi et al., 2019). We used Bonferroni correction methods to adjust all *p*-values [*adjusted p*-value = raw *p-*value ^∗^14 (number of tests)]. All analyses were conducted using R software (R ver. 3.52).

Also, we performed a permutation multiple regression analysis to investigate the relationship between changes in cognitive functions and changes in mood state. The ages were used as covariates. We performed permutation multiple regression analyses using the imputed datasets with the “lmp” function in lmperm package. Significance was inferred for *p* < 0.05.

## Results

There was no significant group difference between the two groups at baseline (Table 1). Five participants dropped out after randomization because of their respective schedules. Following the intention-to-treat rule, we imputed missing values of the five participants (please see the section “Analysis”). %MRMax in the combination exercise group showed about 71%. It meant that the intensity of the combination exercise was the moderate intensity (64%HRMax–74%HRMax) (American College of Sports Medicine, 2013).

To check the benefits of the combination exercise training on cognitive functions and mood state, we performed 2 (Intervention group) by 2 (Age category) permutated ANCOVAs for the change scores (Table 2). Regarding cognitive abilities, we found no significant interaction effects and no significant main effects on any performances. However, the significant main effects of the intervention group on inhibition performances were duly noted. Compared to control group, the combination exercise group showed significant improvements in inhibition performances measured by rST [*F*(1,58) = 10.91, η*^2^* = 0.11, *adjusted p* = 0.023] and ST [*F*(1,58) = 11.69, η*^2^* = 0.11, *adjusted p* = 0.016]. Regarding the mood state, we only found significant main effects of the group on the V–A score [*F*(1,58) = 14.73, η*^2^* = 0.17, *adjusted p* = 0.004]. There were no significant interactions and main effects of age in all measurements.

**TABLE 2:** Changed scores of cognitive functions and mood states of both groups.

	**Combination exercise group**				**Control group**				**2 (group) by 2 (age category) ANCOVA**		
	**Middle age**		**Older**		**Middle age**		**Older**		Adjusted *p*-value* (raw *p*-value)		
	**Mean**	**SD**	**Mean**	**SD**	**Mean**	**SD**	**Mean**	**SD**	**Group effect**	**Age-category effect**	**Interaction effect**
**Cognitive functions**											
Cd	4.14	7.84	3.40	7.89	3.25	6.19	4.57	6.66	1.00	1.00	1.00
									(0.86)	(0.72)	(0.47)
rST	6.00	3.80	4.60	4.67	2.73	2.69	2.08	4.29	0.02	0.32	1.00
									(0.00)	(0.02)	(0.54)
ST	5.79	4.02	5.40	5.54	2.67	2.72	1.92	4.16	0.02	1.00	1.00
									(0.00)	(0.51)	(0.75)
LFT	0.79	3.04	0.33	2.72	1.38	2.99	0.00	3.31	1.00	1.00	1.00
									(0.78)	(0.34)	(0.50)
Updating	0.86	2.18	−0.20	1.82	0.50	2.85	0.21	2.19	1.00	1.00	1.00
									(0.84)	(0.77)	(0.42)
D-CAT	11.29	33.01	11.33	23.82	11.88	23.03	8.14	14.19	1.00	1.00	1.00
									(0.90)	(0.72)	(0.68)
**Mood**											
AH	−1.79	2.69	−1.33	1.68	−2.00	2.58	0.36	2.27	1.00	1.00	1.00
									(0.36)	(0.14)	(0.08)
CB	−0.29	3.12	−1.27	2.12	−0.38	2.28	0.50	2.98	1.00	1.00	1.00
									(0.24)	(0.99)	(0.63)
DD	−0.71	2.02	−1.13	2.42	−1.06	3.15	0.64	1.28	1.00	1.00	0.53
									(0.32)	(0.29)	(0.04)
FI	0.71	5.15	−0.93	2.46	−0.50	2.48	0.07	3.41	1.00	1.00	1.00
									(0.92)	(0.14)	(0.33)
TA	−0.57	1.95	−1.93	2.55	−2.19	3.94	−0.57	1.28	1.00	1.00	0.41
									(0.88)	(0.95)	(0.03)
VA	1.93	2.73	1.53	3.07	−1.38	2.31	−0.36	2.56	0.00	1.00	1.00
									(0.00)	(0.68)	(0.35)
F	0.79	2.69	1.80	2.60	−0.63	2.16	0.57	2.90	0.46	1.00	1.00
									(0.03)	(0.16)	(0.93)
TMD	−3.79	12.04	−6.33	8.61	−5.38	11.26	1.93	7.38	1.00	1.00	1.00
									(0.23)	(0.21)	(0.13)

*SD, standard deviation; Cd, digit symbol coding; rST, reverse Stroop task; ST, Stroop task; LFT, letter fluency task; Updating, working memory updating task; D-CAT, digit cancellation task; AH, Anger–Hostility; CD, Confusion–Bewilderment; DD, Depression–Dejection; FI, Fatigue–Inertia; TA, Tension–Anxiety; VA, Vigor–Activity; F, Friendliness; TMD, total mood disturbance. Means and SDs were the pooled means and SD after multiple imputations (*m* = 20). ^∗^*P*-values were adjusted using Bonferroni correction method. If an adjusted *p*-value was over 1.00, then we use 1.00. Raw *p*-values were shown in parentheses.*

**FIGURE 2:**
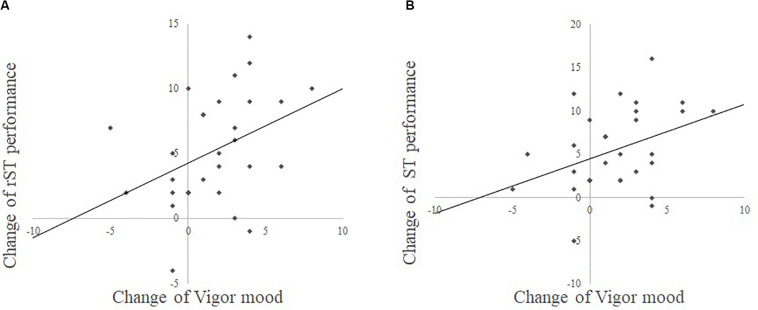
**(A)** Is the scatter plot between the change of rST (Stroop task) performance and the change of Vigor mood from POMS (Profile of Mood State). **(B)** Is the scatter plot between the change of ST performance and change of Vigor mood from POMS.

Additionally, to investigate the relationships between changes in cognitive functions and mood, we separately performed multiple regression analyses in each group. The results of the combination exercise group showed a significant positive correlation between improved rST and improved V–A mood scores (*r* = 0.39, *standardized*β = 0.432, *t* = 2.45, *p* = 0.018, Figure 2A) and between improved ST and improved V–A mood scores (*r* = 0.38, *standardized*β = 0.436, *t* = 2.52, *p* = 0.019, Figure 2B). There were no significant correlations between improved cognitive functions and other mood scores. Moreover, no significant result was found in the multiple regression analyses of the control group.

## Discussion

This study investigated the acute benefits of the single bout of combination exercise training on cognitive functions and mood states in middle-aged and older females. We found three cardinal findings. First, the combination exercise showed significant improvements in inhibition performance measured by the Stroop and reversed Stroop tasks in middle-aged and older females. Second, the combination exercise group demonstrated improvements in the VA mood measured by the POMS in the middle-aged and older females. Finally, a significant positive correlation was found between the change score of inhibition performance and the change score of VA. These findings are discussed below.

The first main finding is that the 30-min combination exercise improved inhibition performances in executive functions. The current result is consistent with previous acute exercise studies. The previous studies reported that a single bout of aerobic exercise improved inhibition performance (Hyodo et al., 2012; Byun et al., 2014). Additionally, a recent study demonstrated that high-intensity aerobic interval training with resistance training had acute benefits on the inhibition performances of young overweight men (Quintero et al., 2018). However, this study is the first demonstrating the improvement of inhibition performance after the single bout of the combination exercise in the healthy middle-aged and older female adults. Besides, the previous study demonstrated that long-term combination exercise training intervention improved inhibition performances (Nouchi et al., 2014; Vaughan et al., 2014). Altogether, in the current and previous findings, the single bout and the long-term combination exercise would have a positive effect on inhibition performances of executive functions.

In this study, we did not find any significant changes in shifting, updating, and processing speed performances after the single bout of the combination exercise training. A few previous studies investigated the acute benefit of a combination exercise on cognitive function. These studies did not use a wide range of cognitive function measures. Therefore, it is difficult to conclude that the combination exercise does not have positive effects on updating and processing speed. A previous meta-analysis study analyzing long-term exercise showed that combination exercises are beneficial for diverse cognitive functions compared to aerobic or strength exercises alone (Barha et al., 2017). The combination exercises would still have a possibility to improve a wide range of cognitive functions. In the future, it is essential to investigate whether the combination exercise regimes have benefits on diverse cognitive functions.

The second main finding is that the VA mood increased after a single bout of the combination exercise training. This result is consistent with the meta-analysis result (Loy et al., 2013). The meta-analysis reported that the single bout of aerobic exercise improved energy and decreased fatigue (Loy et al., 2013). Furthermore, one previous study using the combination exercise training for young females showed that participants felt more energy measures by PANAS (Positive and Negative Affect Schedule) (Watson et al., 1988) after the 1-h combination exercise (Maraki et al., 2005). This study expanded the previous evidence to show that 30 min of combination exercise improved VA in middle-aged and older females.

The third main finding is that the improvements in inhibition performance were positively correlated with the improvement of VA. This result is consistent with previous findings. For example, previous studies employing aerobic exercise showed a positive correlation between the improvement of cognitive functions processing speed/visual attention measured by Trail making test A and the improvement of vigor mood measured by POMS in young adults (Legrand et al., 2018) and older adults (Legrand et al., 2016). Also, another study using aerobic exercise reported a positive correlation between the improvement of inhibition performance measured by the Stroop task and improvement of arousal mood measured by the Two-Dimensional Mood Scale (TDMS) in healthy young adults (Byun et al., 2014). However, this is the first study that demonstrated a positive correlation between the improvement of cognitive performances and the improvement of vigor mood after combination exercise for middle-aged and older females. It is important to consider the exercise intensity on cognition functions and mood states. The exercise intensity of the combination exercise was the moderate level. The current results of improvements of inhibition performance and vigor mood are consisted with the previous evidence. Previous studies demonstrated that the moderate intensity combination exercise improved Stroop performance (Chang et al., 2017) and positive mood (Maraki et al., 2005) in healthy young adults. But, this study firstly demonstrated the moderate intensity combination exercise increase cognitive performance and mood state in middle-aged and older females. It is still unclear that differences of the exercise intensity in a combination exercise on cognitive functions and mood states. In the future study, it should investigate an effect of the exercise intensity in a combination exercise on cognition and mood states.

Although our study did not directly measure the dopamine level, we hypothesize that the dopamine level could provide a mechanistic explanation of the observed benefits. It has shown that dopamine has an imperative role in executive function (Nieoullon, 2002) and motivational mood states (Badgaiyan et al., 2009; Takeuchi et al., 2017). Interestingly, the dopamine did not equally affect all components of the executive functions (Zhang et al., 2015). Previous pharmacological studies reported that the change of the dopamine level was significantly associated with inhibition performances in animals and humans (Devito et al., 2009; Eagle et al., 2011). Previous animal and human studies demonstrated that a single bout of exercise affected dopaminergic systems (Basso and Suzuki, 2016). Animal studies showed that dopamine significantly increased in the brain after a single bout of exercise (Meeusen and De Meirleir, 1995). Human studies also reported that the acute exercise changed the peripheral dopamine levels (Skriver et al., 2014) and brain activities in the dopaminergic brain area, such as reward-related and prefrontal regions (Li et al., 2014; Weng et al., 2016). It is a possibility that other changed of neurotransmitter such as serotonin or neurotrophins [e.g., BDNF and insulin-like growth factor 1 (IGF-1)] (Basso and Suzuki, 2016). Therefore, future studies should check the change of neurotransmitters or neurotrophins, as putative mechanisms underlying the observed acute benefit after the combination exercise.

This study has some limitations. First, to reduce the effects of physical exercise experience, we recruited only female participants. However, previous studies reported varying exercise benefits for both sexes. The female participants received more benefits from physical exercise (Barha et al., 2017). It is crucial to investigate whether the combination exercise has acute benefits in the male participants and whether the gender differences in the acute benefit derived from combination exercise regimes exist. Second, we used the passive control group which did not do any exercise. Most acute exercise studies (Yeung, 1996; Chang et al., 2012; Barha et al., 2017; Elkington et al., 2017; Levin et al., 2017) used a similar passive control group. Studies using the passive control group would give a message for people who do not have exercise habits due to lack of time. The message which a single and short exercise improves cognitive function and mood state could enhance to start exercise and make a habit of doing regular exercise. However, it is also important to investigate whether the combination exercise has more benefits on cognitive functions and mood states compared to other exercises such as walking or yoga. In future studies, the use of an active control group is recommended.

## Conclusion

We investigated the acute benefits of the combination exercise on diverse cognitive functions and mood states in middle-aged and older females. Our study demonstrated that a 30-min single bout of the combination exercise improves inhibition performances and the VA mood in middle-aged and older females.

## Data Availability Statement

The datasets generated for this study are available on request to the corresponding author.

## Ethics Statement

The studies involving human participants were reviewed and approved by Institutional Review Board of the Tohoku University Hospital. The patients/participants provided their written informed consent to participate in this study.

## Author Contributions

RN designed and developed the study protocol and analyzed all the data. RN and HN conducted the study. RN, HN, and RK wrote the manuscript. RK provided advice related to the study protocol. All authors have read and approved the final manuscript.

## Conflict of Interest

The authors declare that the research was conducted in the absence of any commercial or financial relationships that could be construed as a potential conflict of interest.

## Footnotes

1 http://www.consort-statement.org/home/

2 http://www.graphpad.com/quickcalcs/index.cfm

3 http://cran.r-project.org/web/packages/lmPerm/index.html
